# Supplementary material for: Multivariate Independent Component Analysis Identifies Patients in Newborn Screening Equally to Adjusted Reference Ranges
Source: Int J Neonatal Screen. 2023 Oct 20;9(4):60. doi: 10.3390/ijns9040060 (PMC10594528; doi:10.3390/ijns9040060)
Supplement: Supplementary file 1 [file IJNS-09-00060-s001.zip › Table S1 List of IEMs.pdf]

Table S1 - Complete list of OMIM numbers, names of diseases, and their incidence

Disease	Full name	OMIM	Incidence
PKU/HPA	Phenylketonuria/ Hyperphenylalaninemia	261600/ 233910	1 : 6 500
MSUD	Maple syrup urine disease	248600	1: 185 000
MCAD	Medium-chain acyl-CoA dehydrogenase deficiency	201450	1 : 18 712
LCHAD	Long-chain 3-hydroxyacyl-coenzyme A dehydrogenase deficiency	609016	1 : 56 136
VLCAD	Very long-chain acyl-CoA dehydrogenase deficiency	201475	1 : 280 680
CPT I	Carnitine palmitoyl transferase I deficiency	255120, 616282	rare
CPT II/CACT	Carnitine palmitoyl transferase II deficiency/ Carnitine-acylcarnitine translocase deficiency	255110, 608836, 600649, 614212/ 212138	rare
GA I	Glutaric aciduria I	231970	1 : 40 000
IVA	Isovaleric acidemia	243500	1 : 230 000
HCY (CBS)	Homocystinuria due to cystathioine beta-syntase deficiency	236200	1 : 100 000
HCY (MTHFR)	Homocystinuria due to deficiency of N(5,10)-methylenetetrahydrofolate reductase activity	236250	rare
ARG	Argininemia	207800	1 : 300 000
CIT/ASA	Citrullinemia/ Argininosuccinic aciduria	215700/ 207900	1 : 78 000

Source: Novorozenecký screening. [cited 20 Feb 2023].
Available: https://www.novorozeneckyscreening.cz/
