# Supplementary figures and images for: Multivariate Independent Component Analysis Identifies Patients in Newborn Screening Equally to Adjusted Reference Ranges
Source: Int J Neonatal Screen. 2023 Oct 20;9(4):60. doi: 10.3390/ijns9040060 (PMC10594528; doi:10.3390/ijns9040060)

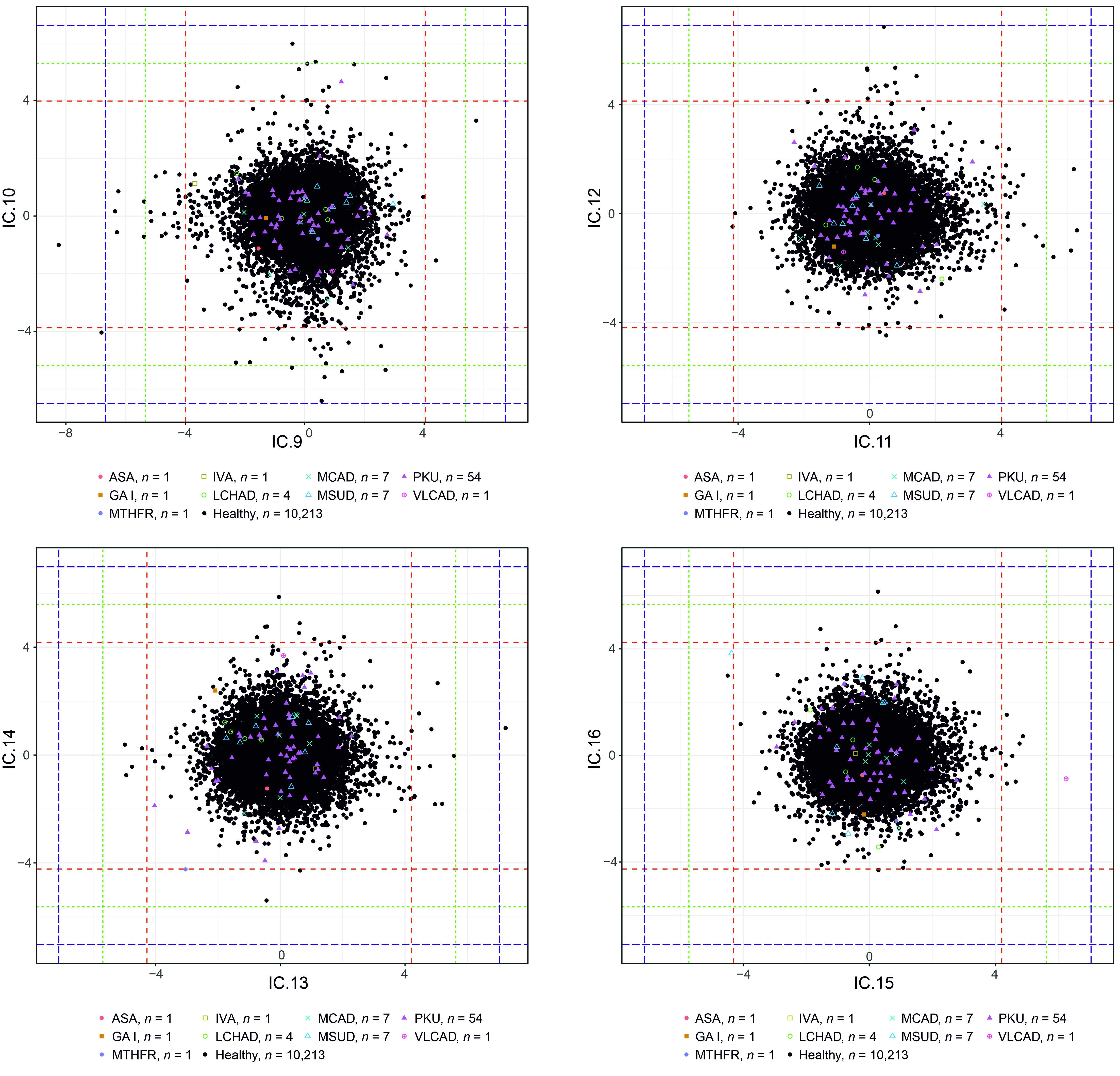

Supplement: Supplementary file 1 [file IJNS-09-00060-s001.zip › Fig. S1 Score plots IC9-16 discovery study.jpg]

Scree plot ICA

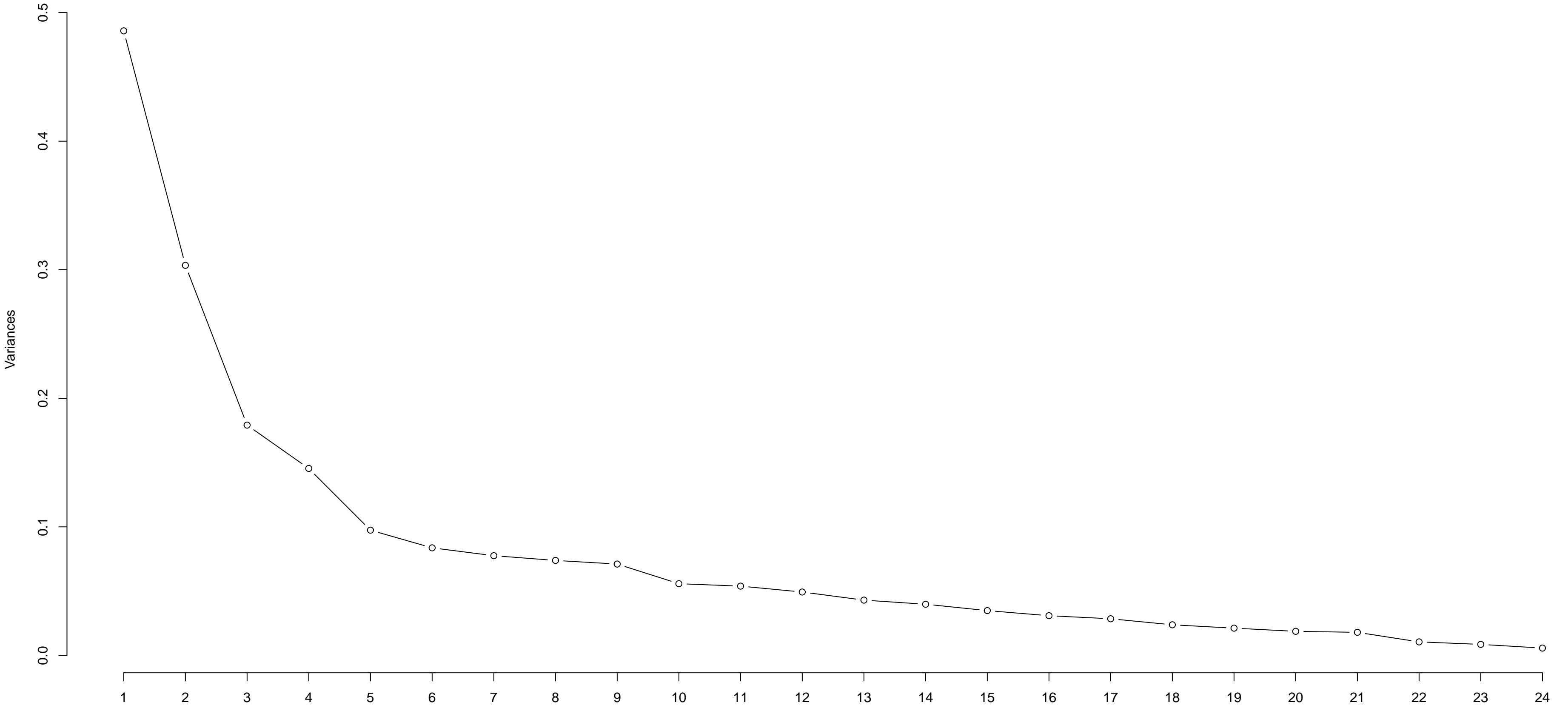

Supplement: Supplementary file 1 [file IJNS-09-00060-s001.zip › Fig. S3 PCA scree plot.pdf]

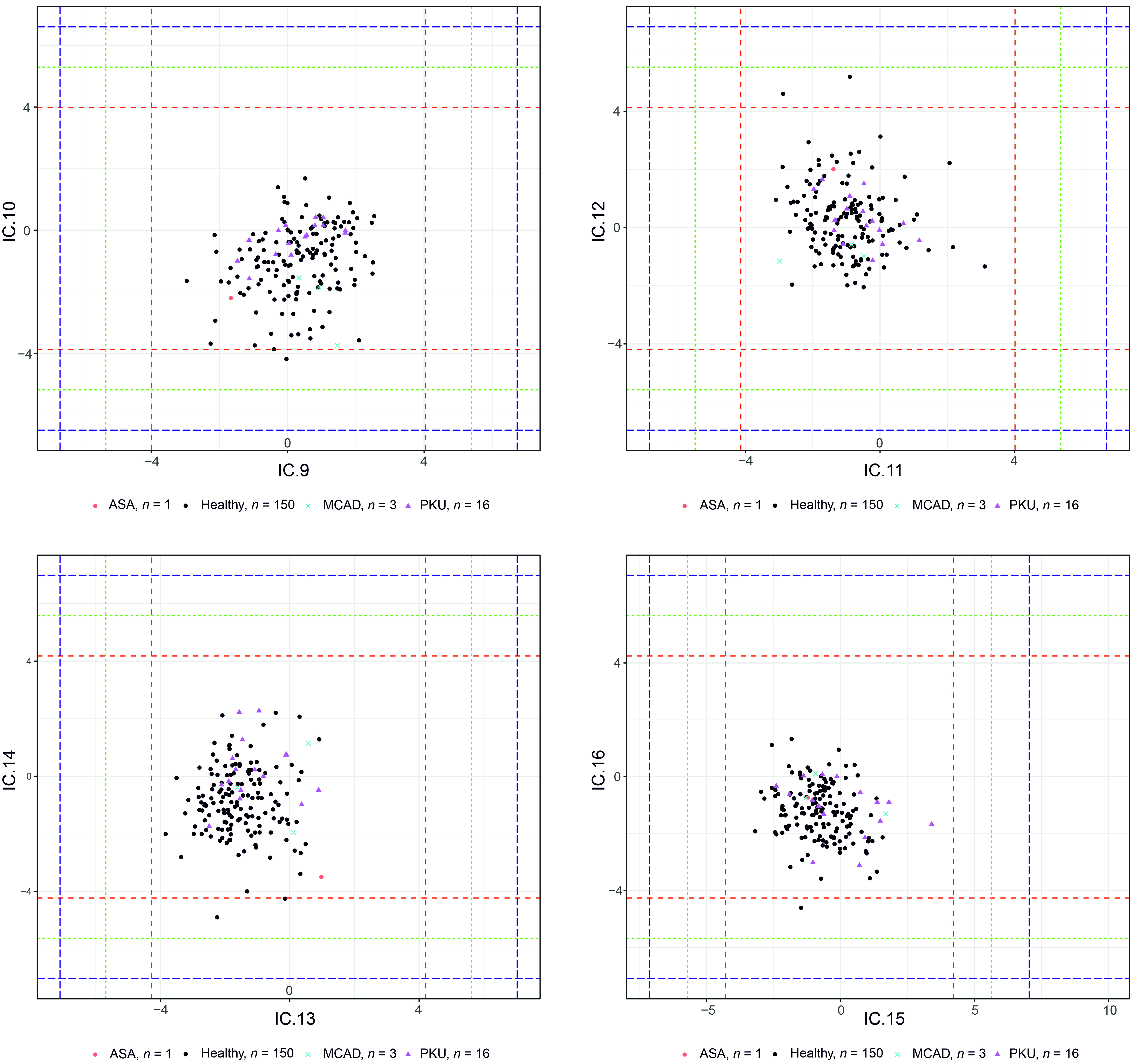

Supplement: Supplementary file 1 [file IJNS-09-00060-s001.zip › Fig. S4 Score plots IC9-16 validation study.jpg]
